# A 3D-printed microfluidic-enabled hollow microneedle architecture for transdermal drug delivery

**DOI:** 10.1063/1.5127778

**Published:** 2019-12-11

**Authors:** Christopher Yeung, Shawnus Chen, Brian King, Haisong Lin, Kimber King, Farooq Akhtar, Gustavo Diaz, Bo Wang, Jixiang Zhu, Wujin Sun, Ali Khademhosseini, Sam Emaminejad

**Affiliations:** 1 Interconnected & Integrated Bioelectronics Lab (I_2_BL), Department of Electrical and Computer Engineering, University of California, Los Angeles, California 90095, USA; 2 Department of Materials Science and Engineering, University of California, Los Angeles, California 90095, USA; 3 Department of Bioengineering, University of California, Los Angeles, California 90095, USA; 4 Center for Minimally Invasive Therapeutics (C-MIT), California NanoSystems Institute, University of California, Los Angeles, California 90095, USA; 5 Department of Radiology, University of California, Los Angeles, California 90095, USA; 6 Department of Chemical and Biomolecular Engineering, University of California, Los Angeles, California 90095, USA; 7 Jonsson Comprehensive Cancer Center, University of California, Los Angeles, California 90095, USA

## Abstract

Embedding microfluidic architectures with microneedles enables fluid management capabilities that present new degrees of freedom for transdermal drug delivery. To this end, fabrication schemes that can simultaneously create and integrate complex millimeter/centimeter-long microfluidic structures and micrometer-scale microneedle features are necessary. Accordingly, three-dimensional (3D) printing techniques are suitable candidates because they allow the rapid realization of customizable yet intricate microfluidic and microneedle features. However, previously reported 3D-printing approaches utilized costly instrumentation that lacked the desired versatility to print both features in a single step and the throughput to render components within distinct length-scales. Here, for the first time in literature, we devise a fabrication scheme to create hollow microneedles interfaced with microfluidic structures in a single step. Our method utilizes stereolithography 3D-printing and pushes its boundaries (achieving print resolutions below the full width half maximum laser spot size resolution) to create complex architectures with lower cost and higher print speed and throughput than previously reported methods. To demonstrate a potential application, a microfluidic-enabled microneedle architecture was printed to render hydrodynamic mixing and transdermal drug delivery within a single device. The presented architectures can be adopted in future biomedical devices to facilitate new modes of operations for transdermal drug delivery applications such as combinational therapy for preclinical testing of biologic treatments.

## INTRODUCTION

Advancements in bioengineering and materials science have accelerated the progression toward personalized medicine by creating technologies that facilitate disease diagnosis and delivery of care at the “point-of-person.”^1–3^ Among the many technologies critical to the realization of personalized medicine, transdermal drug delivery is one of the most important, because it enables safe, painless, and convenient drug delivery.^4^ In this regard, microneedles provide promising solutions for transdermal drug delivery because of their minimally invasive nature. Microneedles were previously constructed with a wide variety of materials including metals, ceramics, and polymers.^5–7^ Among these materials, biocompatible polymer microneedles have undergone widespread adoption over metal or ceramic microneedles due to their ability to be manufactured at low costs, safely disposed after use, and tailored for customized drug release needle profiles.^8^

The diversity and versatility of operations enabled by microneedles can be augmented through the integration of microfluidic devices to achieve new drug delivery capabilities.^9–11^ Specifically, the ability of microfluidics to rapidly transport and mix small volumes of fluids, while varying reaction conditions (e.g., flow rates and reactant concentrations) to optimize the resulting products, makes it an effective technology to deliver numerous operations relevant to transdermal drug delivery.^12^ For example, microfluidic mixing was used to directly synthesize nanoparticles with tunable physicochemical properties such as particle size, homogeneity, and drug loading and release at the point of delivery.^13^ Additionally, the combination of microneedles and microfluidic mixing is beneficial in areas such as combinational therapy-based subcutaneous/transdermal administration for preclinical testing of biologic treatments.^14^ As more treatments are developed for codosing, where patients receive multiple treatments simultaneously, the availability of subcutaneous/transdermal alternatives could reduce treatment complexity and costs, allowing flexibility in combining different biologics without extensive formulation development.^14^

Conventional fabrication schemes are not well-suited to realize both microneedles and microfluidic structures within a single device, because they cannot efficiently and/or simultaneously realize features that operate at two distinct length-scale domains. Operationally, epidermal penetration for enhanced drug permeability requires micrometer-scale microneedles,^15^ while microfluidic channels route and manipulate microliters of fluid within millimeter/centimeter-long devices.^16^ As a result, previous work commonly relied on the fabrication of microneedles and microfluidic channels as disjointed modules, which were individually constructed via separate, costly, and multistep techniques.^23^ For instance, microneedle fabrication processes consist of micromolding, electrodeposition, mold-based etching, and/or solvent casting,^19–22^ while microfluidic channel fabrication processes include laser-cutting, micromilling, and/or lithography.^17,18^ Therefore, a suitable alternative is preferred, which is capable of rendering both types of structures with both the required resolution for epidermal penetration and higher-throughput manufacturing.

Accordingly, 3D-printing is a suitable alternative to conventional fabrication methods because, in principle, it allows the realization of customizable and intricate microfluidic/microneedle architectures without the commonly required investments.^23^ However, previously reported 3D-printed microchannel-microneedle platforms relied on expensive two-photon polymerization (2PP) 3D-printers to print microneedles and required additional processes to integrate with separately fabricated microchannels.^24^ While 2PP offers high print resolutions down to the 200 nm regime, studies have shown that microneedle tips with ∼30 *μ*m radius of curvature are enough for epidermal penetration.^25^ Furthermore, the small build volume (8 mm maximum object height) and long print times (e.g., ∼3 h for a 1 × 1 × 6 mm^3^ spring) associated with 2PP machines make the technology impractical for rendering larger features such as millimeter/centimeter-long microfluidic channels.^26^

Here, we present a rapid, low-cost, and single-step stereolithography (SLA)-based 3D-printing scheme to simultaneously render multiscale features (from micrometers to centimeters) and unify them into microfluidic-enabled microneedle devices. SLA printers offer the ability to simultaneously and efficiently print submillimeter/centimeter-scale components with larger build volumes (∼20–30 cm maximum height), shorter print times (e.g., ∼15 min for the equivalent 6 mm tall spring^26^), and substantially higher throughput than 2PP printers without any compromise in device performance. These operational advantages, combined with the lower cost and weight of commercially available SLA printers,^27,28^ make them viable for field deployment in lower resource settings, where transdermal drug delivery devices can be rapidly prototyped and tailored toward an individual patient's needs.

To harvest the benefits of SLA-based fabrication schemes for envisioned applications, the limitations of standard SLA printers must be overcome to reach the resolutions required for optimal microneedle performance (e.g., in terms of ease of penetration and minimized pain). In this regard, we devised an elaborate hollow microneedle design and a refined print setup to produce needle tips substantially finer than the SLA printer's laser spot size and with high yield. To demonstrate the utility of the presented microfluidic/microneedle fabrication approach, we 3D-printed an architecture consisting of a hydrodynamic mixing module interfaced with a hollow microneedle array. This architecture allows the modulation of the input fluid solutions' flow rates to facilitate programmable drug delivery in future combinational therapy-based applications.

## MATERIALS AND METHODS

### Microchannel-microneedle fabrication and design

The microfluidic-enabled microneedle device was fabricated using an SLA printer (Formlabs, Form 2) and class IIa biocompatible resin (Formlabs, Dental LT Clear). The resin is a mixture of methacrylic acid esters and photoinitiator comprised of (in % w/w) >70% methacrylic oligomer, <20% glycol methacrylate, <5% pentamethyl-piperidyl sebacate (EG No. 255-437-1), and <5% phosphine oxide (EG No. 278-355-8).^50^ It possesses high optical transparency for visual characterization and flexural strength greater than 50 MPa and supports a print resolution of 100 *μ*m. The device was designed in computer-aided design (CAD) software (Autodesk, Fusion 360) and preprocessed in 3D-printer preparation software (Formlabs, PreForm). Within PreForm, 0.6 mm touch point sizes were automatically generated at the default support density. Supports were adjusted to ensure they were not arranged near critical features such as the microchannel inlets and hollow microneedles (4 × 4 array). To optimize microneedle yield and quality, the model was oriented at 45° to the build platform. Following SLA printing, the printed parts were washed in isopropyl alcohol (IPA, 99.9% v/v) for 5 min (Formlabs, FormWash) and cured in a 405 nm wavelength UV chamber at 80 °C for 20 min (Formlabs, FormCure) to maximize stiffness and strength.

### Microneedle penetration and mechanical characterization

Penetration tests were performed with parafilm (8 layers, ∼1.1 mm total thickness) and a 500 g weight (∼5 N). The microneedles were placed on top of the parafilm, onto which the weight was applied for 1 min. The microneedles were then removed and the parafilm unfolded to examine the depth of penetration. Microneedle fracture tests were conducted on the syringe-shaped microneedle arrays using an Instron 5943. Arrays were placed needle-side up on the stage and compressed with a 10 kN load cell at 1 mm/min up to 50 N of applied force. After five independent trials, the mean forces and standard deviations (±SDs) were calculated. 0.4% wt/vol trypan blue in phosphate-buffered saline (PBS; Sigma Aldrich) was used for porcine skin (Stellen Medical, 1.5 mm thick) penetration testing to stain the perforation sites.

For mechanical simulation (Autodesk, Fusion 360), a 0.05 N force was applied to a single needle tip to emulate the force per needle applied from the penetration tests. The cylindrical extrusion at the tip of the needle was replaced with a half-sphere to replicate the final printed structure.

### Microfluidic characterization

Microfluidic tests were performed with dye solutions (Adam's Extract) and programmable syringe pumps (Harvard Apparatus, Pump 11 Pico Plus and PHD ULTRA). The syringes were pumped at four flow rate ratios (1:1:1, 0.1:0.1:1, 0.1:1:0.1, and 1:0.1:0.1) corresponding to the flow rates of the red-dyed, clear water, and blue-dyed solutions (Q_1_:Q_2_:Q_3_). To visualize hydrodynamic mixing characteristics, the following flow rates were applied. For the 1:1:1 baseline flow rate, 100 *μ*l/min was applied to all three inlets. For the 0.1:0.1:1, 0.1:1:0.1, and 1:0.1:0.1 flow rates, 10 *μ*l/min and 100 *μ*l/min were used for 1 and 10 flow rate ratios, respectively.

Three fluorochrome-water solutions were used for concentration measurements: rhodamine B, fluorescein isothiocyanate, and methylene blue (Sigma Aldrich) at 0.5% wt/vol concentrations. Fluorescent dye concentrations were measured with 25 *μ*l sample volume in a 384-well microplate (Thermo Fisher Scientific) and a microplate reader (Tecan, Infinite M1000 Pro). Excitation wavelengths of 545 nm, 489 nm, and 664 nm, and emission wavelengths of 566 nm, 517 nm, and 690 nm were used for rhodamine B, fluorescein isothiocyanate, and methylene blue, respectively.

### Porcine skin fluorochrome delivery and image analysis

Porcine skin was cut into 2 × 2 cm pieces and the same fluorochrome-water solutions used for microfluidic characterization were used for model-drug delivery tests. The combined spiral microfluidic and microneedle device was pressed onto the porcine skin, and the fluorochrome solutions were injected with the aid of the syringe pumps. Identical instrumentation and flow rate ratios from the microfluidic characterization were used in these tests (where the absolute flow rates were reduced by a factor of 10 to minimize leakage).

After removing the device, images of the porcine skin penetration area were captured using confocal microscopy (Figs. S5–S8 in the supplementary material), and the raw fluorescence intensities of all images were quantified using ImageJ software. This process was repeated for each fluorochrome to obtain their intensities at various skin depths. The integral of the interpolated intensity vs the skin depth curve (yielding total intensity) for each fluorochrome is used to infer the dye amount delivered to the skin. Then, these values were divided by their respective total intensities captured at the 1:1:1 baseline flow rate condition for ease of comparison.

### Scanning electron and confocal laser scanning microscopy

The microneedles and microchannels were imaged using scanning electron microscopy (Hitachi S4700 SEM) with an accelerating voltage of 10 kV. All samples were sputtered with a 7 nm gold coating prior to imaging (Anatech Ltd, Hummer 6.2).

Fluorescence images of each fluorochrome were taken using a confocal laser scanning microscope (Leica, TCS-SP5). For rhodamine B, a 543 nm wavelength HeNe laser was set at 80% intensity and fluorescence between 560 and 640 nm wavelengths were captured. For fluorescein isothiocyanate, 496 and 488 nm wavelength argon lasers were both set at 100% and fluorescence between 510 and 560 nm wavelengths were captured. Lastly, for methylene blue, a 633 nm HeNe laser was set at 25% intensity and fluorescence between 650 and 750 nm wavelengths were captured. Smart gain was 700 across all samples, and five images were taken at 100 nm steps across 0–400 *μ*m skin depths (0 *μ*m represents the skin surface).

## RESULTS AND DISCUSSION

### Microneedle fabrication with SLA 3D-printing

**FIG. 1.**
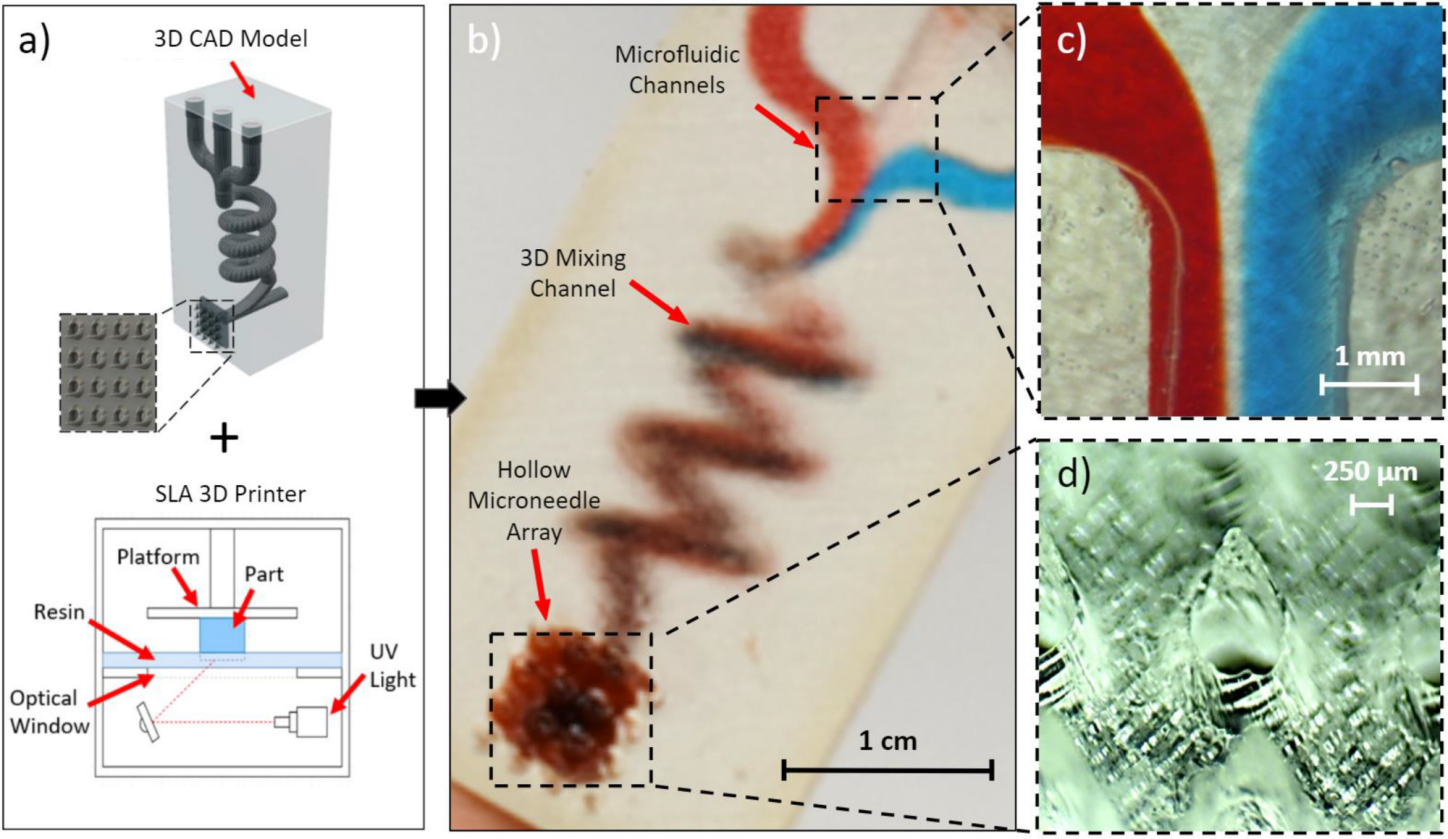
3D-printing of microfluidic-enabled hollow microneedle devices. (a) CAD model of a representative microfluidic-enabled microneedle device as an input to the SLA printer. (b) The printed device with three microfluidic inlets converging into a 3D spiral chamber and to a hollow microneedle array outlet. (c) Close-up of the inlet junction visualizing the convergence of red-dyed, clear, and blue-dyed solution streams. (d) Close-up of the hollow microneedle array.

As conceptualized in Fig. 1(a), we leveraged SLA 3D-printing to fabricate a single-piece, multi-inlet, 3D microfluidic device with an embedded hollow microneedle array. The utilized printer directs UV light through an optical window below the build platform and additively manufactures the designed model by curing a photopolymer resin layer-by-layer within the resin tank. Figure 1(b) showcases a representative postprinted device and highlights its core microfluidic and microneedle components: an illustrative 3D hydrodynamic mixing microfluidic module and a syringe-shaped microneedle array. Final device dimensions are 1.5 × 1.2 × 3.1 cm^3^, and 12 devices can be created in 2.5 h in a single print (effectively yielding 12.5 min per device). Furthermore, the material selected for device fabrication (Formlabs, Dental LT Clear) is a class IIa biocompatible resin, which belongs to a category of materials previously shown to be suitable for transdermal applications.^29,33^

### Investigation and optimization of microneedle design

To optimize for penetration performance, we investigated various microneedle geometries while following previously established design guidelines, which require the width, bore, and length of the microneedles to be approximately 1 mm.^30,31^ In accordance with previous studies, we also minimized the radius of curvature of the needle tips to reduce pain and insertion force, thereby promoting ease of penetration for the average patient.^30^

**FIG. 2.**
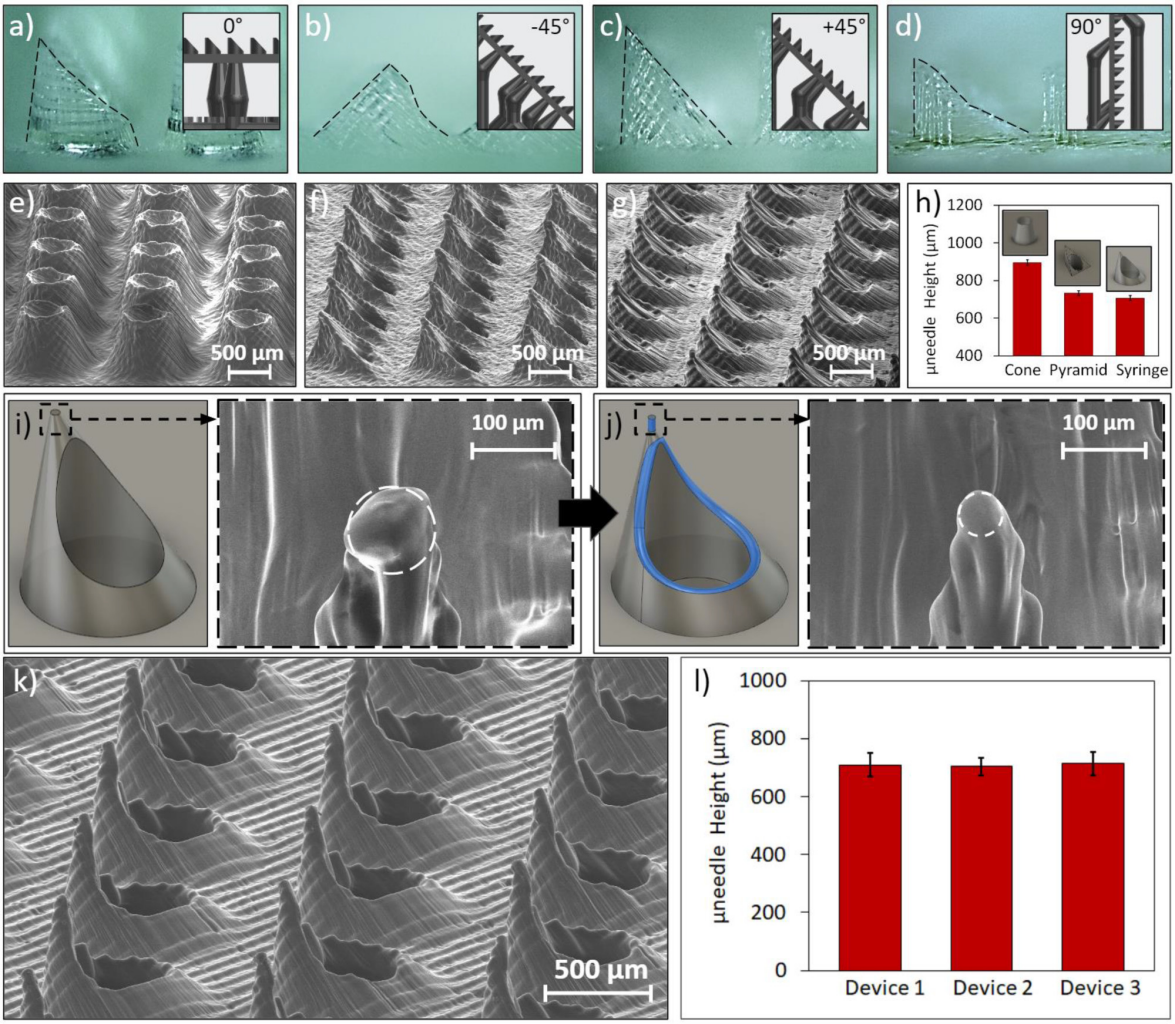
Characterization of 3D-printed hollow microneedle arrays. Images of sheared-cylinder microneedles printed at (a) 0°, (b) −45°, (c) +45°, and (d) 90° angles with outlined profiles (insets show the corresponding print setups). SEM images of the (e) conical, (f) pyramidal, and (g) basic syringe-shaped needle arrays. (h) Average needle heights for each design (for a subset of 25 microneedles per array). (i) CAD model of the syringe-shaped design and the SEM image of the tip (∼50 *μ*m radius of curvature). (j) CAD model of the fine-tip syringe-shaped design (additional features highlighted) and the SEM image of the tip (∼25 *μ*m radius of curvature). (k) SEM image of the fine-tip microneedle array. (l) Average microneedle heights across three separate fine-tip microneedle arrays (for a subset of 25 microneedles per array). Error bars indicate ±standard deviation.

To determine the optimal print setup, we printed a basic sheared-cylinder microneedle design (900 *μ*m tall cylinder with a 45° cut) at different orientations (0°, −45°, +45°, and 90° to the print bed). Optical images of microneedles printed at these orientations are found in Figs. 2(a)–2(d) (with outlined profiles) and Fig. S1 in the supplementary material (without outlined profiles). We found that +45° led to the highest microneedle quality, as this orientation allowed the resin flow (caused by build platform movement) to exert less transverse forces on the tip of the needles, thus mitigating structural warpage. Although the 0° orientation did not cause significant microneedle warpage, it required more complex support structures and resulted in less successful prints.

After determining the ideal print angle, we printed hollow microneedles with different geometries (all with the +45° angle) to ascertain the most effective overall design. Each geometry was designed with an 800 *μ*m wide base, 600 *μ*m center bore, and heights below 1 mm. These hollow microneedle interfaces enable transdermal drug delivery through a “poke-and-flow” mode of operation,^32^ where the tip creates micropores in the skin, followed by the delivery of fluids flowing through the hollow microneedle bore. The printed geometries included two of the most commonly reported 3D-printed microneedle designs: a pyramidal microneedle with a triangular base and tip orthogonally aligned with a vertex of the base,^33,34^ and a conical microneedle with tapered sidewalls.^35–37^ In Figs. 2(e) and 2(f), scanning electron microscope (SEM) images of the pyramidal and conical microneedles in 10 × 10 arrays are shown, respectively. Additionally, we devised a third “syringe-shaped” design that adopted an oblique cone structure with sharper tips than the previous designs [Fig. 2(g)]. To assess the consistency of the SLA-printed features, for each array, we characterized and compared the microneedle heights. Figure 2(h) shows that the average height of the conical, pyramidal, and syringe-shaped microneedles was 900 *μ*m, 730 *μ*m, and 710 *μ*m, respectively (error bars indicate ±standard deviation). The small standard deviations (∼10 *μ*m) across all three designs indicate that the devised SLA printing approach is capable of printing large microneedle arrays of the same design with high degrees of consistency.

To achieve ease of penetration, the radius of curvature at the needle tip must be further minimized, well below the SLA printer's full width half maximum (FWHM) laser spot size (140 *μ*m).^40^ Accordingly, as shown in Figs. 2(i) and 2(j), we incorporated auxiliary design features to further enhance the tip sharpness. Specifically, we added a cylindrical element (diameter: 40 *μ*m, height: 80 *μ*m) to the tip to enforce a high height-to-base aspect ratio, which decreases the effect of diffraction leading to “false printing.”^25^ The false printing phenomenon is illustrated in Fig. S10(a) in the supplementary material, where the expected print resolution is the FWHM spot size, but the diffracted light can cure the photopolymer in undesired areas around the spot size. With a low aspect ratio design [Fig. S10(b) in the supplementary material], there is more exposed material around the laser spot, causing undesired curing that propagates through the subsequent layers due to the gradual transition from a large to small cross-sectional area. With a high aspect ratio tip, which eliminates the cross-sectional area transition entirely [Fig. S10(c) in the supplementary material], the final layers are constructed through the vertical stacking of single-point layers, thus reducing the effects of excess material forming around the spot size and allowing the tip to achieve a smaller overall cross-sectional area. By adding this cylindrical element, we rendered fine-tip syringe-shaped microneedles with radii of curvature as low as 25 *μ*m.

To achieve a 100% hole formation success rate without the need for further postprocessing fabrication steps, we added a fillet to the perimeter of the microneedle's bore to increase its opening. As shown in Fig. S11(a) in the supplementary material, without the fillet, we achieved approximately 50% hole formation. After adding the fillet, 100% hole formation rate was obtained [Fig. S11(b) in the supplementary material]. This fillet is shown in Fig. 2(j) as the blue feature along the edge of the microneedle bore, which resembles a blue ring. Figure 2(k) shows an SEM image of the final fine-tip microneedle in a 10 × 10 array. We repeated the needle-height characterization procedure for three separately printed fine-tip microneedle arrays and found average heights of 710 *μ*m, 705 *μ*m, and 710 *μ*m [Fig. 2(l)], demonstrating a high degree of uniformity across multiple arrays.

### Penetration characterization of microneedle designs

**FIG. 3.**
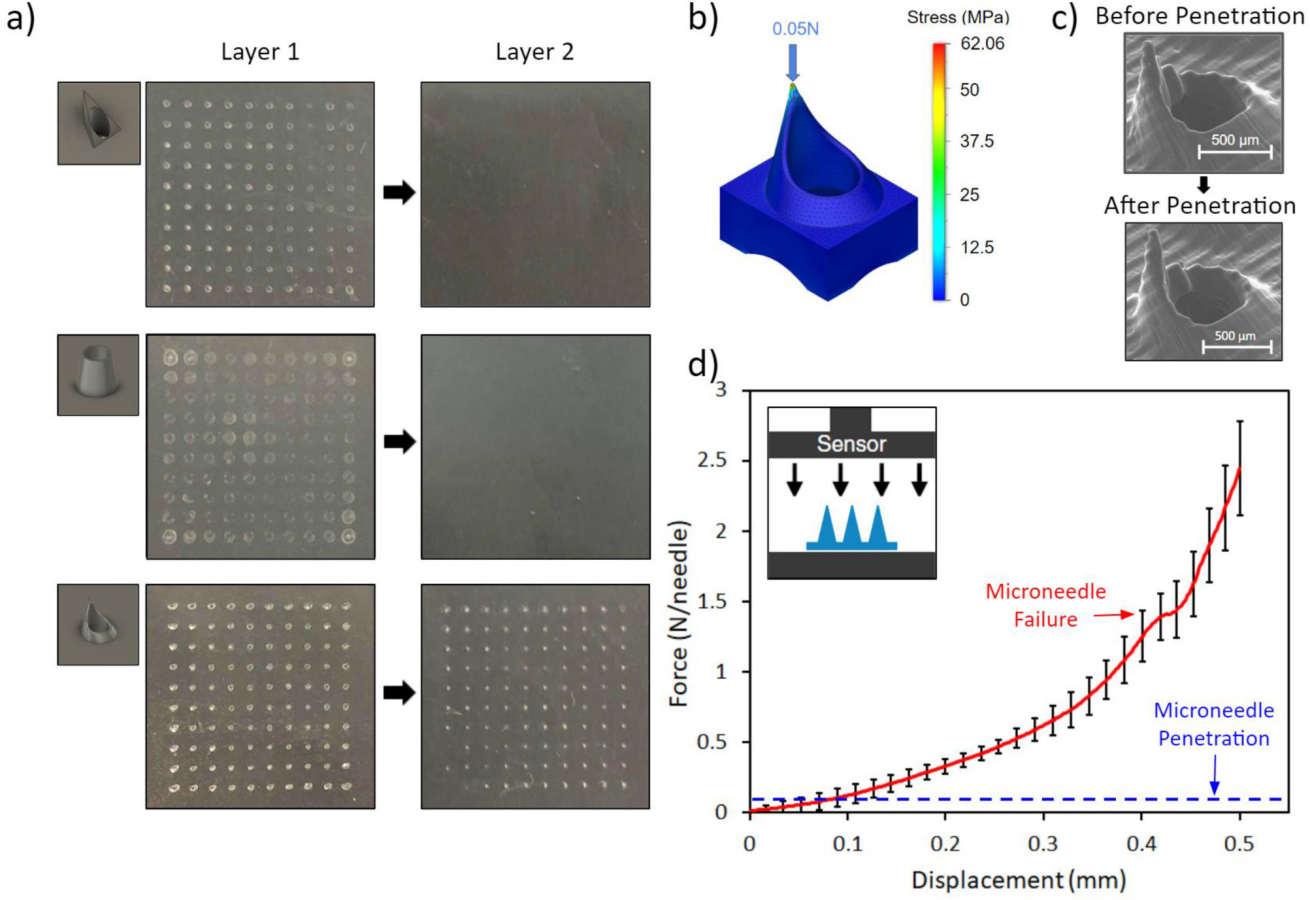
3D-printed microneedle mechanical characterization: penetration and failure. (a) Penetration test of the pyramidal, conical, and fine-tip syringe-shaped microneedle arrays across two layers of the parafilm with 5 N of applied force. (b) Mechanical simulation of the fine-tip syringe-shaped microneedle, visualizing the occurrence of maximal stress at the tip. (c) SEM image of a microneedle before and after penetration testing (demonstrating no tip failure). (d) Axial force vs displacement curve for a 3 × 3 array of syringe-shaped microneedles. The failure point and penetration force are noted. The inset image illustrates the compression test setup.

The performances of the conical, pyramidal, and fine-tip syringe-shaped microneedle designs were evaluated and compared following a previously reported penetration test protocol.^38^ Specifically, penetration was tested on 1.1 mm stacks of parafilm (8 layers), replicating the mechanical properties of skin.^39^ The parafilm technique provides a consistent and convenient method of visually analyzing penetration by overcoming the inconsistent, elastic nature of skin and its tendency to recover following penetration. The microneedle arrays were pressed onto the parafilm stacks with a 500 g weight (5 N, emulating the force applied by an average patient^40^) for 1 min. Then, the first two layers of the parafilm stacks were imaged. To ensure a uniform stress distribution across the parafilm, we sanded the back of the microneedle array to remove residual supporting materials. Figure 3(a) shows the imprint left behind by each microneedle array. The pyramidal microneedles left marks on the first layer of parafilm but not on the second. The conical microneedles merely indented the first layer of parafilm and also did not affect the second layer. The syringe-shaped microneedles successfully penetrated the first layer of parafilm and left behind clear imprints on the second layer, amounting to penetration depths of at least 130 *μ*m (1 parafilm layer thickness).

Based on this penetration study, it can be concluded that the devised syringe-shaped microneedle design maximizes depth and ease of penetration compared to other designs and is capable of comfortably piercing through the human *stratum corneum* (topmost layer of skin, 10–20 *μ*m thickness^41^) without excess force. To further validate the penetration of our syringe-shaped microneedle array, we conducted *ex vivo* porcine skin penetration tests*.* The microneedle arrays were inserted into neonatal porcine skin and removed after 1 min, and then the skin surface was stained with trypan blue (which specifically stains the perforated stratum corneum sites^42^) for 5 min. The corresponding images of the penetration area with and without staining are shown in Figs. S2(a) and S2(b) in the supplementary material. Trypan blue staining confirmed the penetration of the *stratum corneum*, evident from the appearance of blue dots in the skin. Although a few dots are missing at their respective penetration sites, this can be explained by the innate inconsistencies of skin and the subsequent limitations of the trypan blue staining method.^42^

### Syringe-shaped microneedle mechanical characterization

We simulated the stress distribution experienced by the fine-tip syringe-shaped interface to identify the location of the maximum stress (corresponding to microneedle's primary point of failure). Figure 3(b) presents a finite element analysis (FEA) mechanical simulation of the syringe-shaped microneedle (with conditions matching the conducted penetration test), demonstrating the concentration of the incurred stress at the tip of the microneedle. SEM images of a microneedle pre- and postpenetration [Fig. 3(c)] confirm that the microneedle tips do not fail or exhibit noticeable deformation after penetration. Additionally, to assess the mechanical strength of the 3D-printed microneedles, failure tests were conducted on five devices (3 × 3 syringe-shaped microneedle arrays). Following previously reported microneedle compression tests, a uniaxial load was applied directly onto the microneedle tips.^38^ Compression tests [Fig. 3(d)] reveal that failure strength was ∼1.5 N/needle, well above the ∼0.05 N/needle (derived from the force exerted by the 500 g weight onto 100 needles) penetration force, thus validating our microneedle's suitability for on-body applications.

To further evaluate the mechanical robustness of our microneedles and to determine whether they would fracture and leave behind fragments in the skin during/after application, we coated the microneedles with fluorescent dye (0.5% wt/vol rhodamine B for 1 h) and inserted them into porcine skin, then applied a ∼5 N shear force to induce fracture. Fig. S3(a) in the supplementary material shows a fluorescent confocal scanning laser microscope (CLSM) image of the penetration area after the microneedles were removed, revealing mild staining on the skin surface but no indication of microneedle fragments. As a positive control, we deliberately fabricated fragile microneedles (an array of 100 needles, each 700 *μ*m tall and 200 *μ*m wide) and repeated the same fracture test. Fig. S3(b) in the supplementary material shows a CLSM image of the fragile-microneedle penetration sites, containing spots with greater fluorescence intensity than the control study, indicating broken microneedle fragments at these locations. SEM images of the fragile-microneedles before and after shearing [Figs. S4(a) and S4(b) in the supplementary material] illustrate that they primarily exhibit ductile failure and generally remain intact after drastic plastic deformation. Collectively, these results demonstrate that the devised syringe-shaped microneedle array possesses high mechanical stability and do not leave splinters in the skin during/after application.

### Microfluidic modules and mixing

**FIG. 4.**
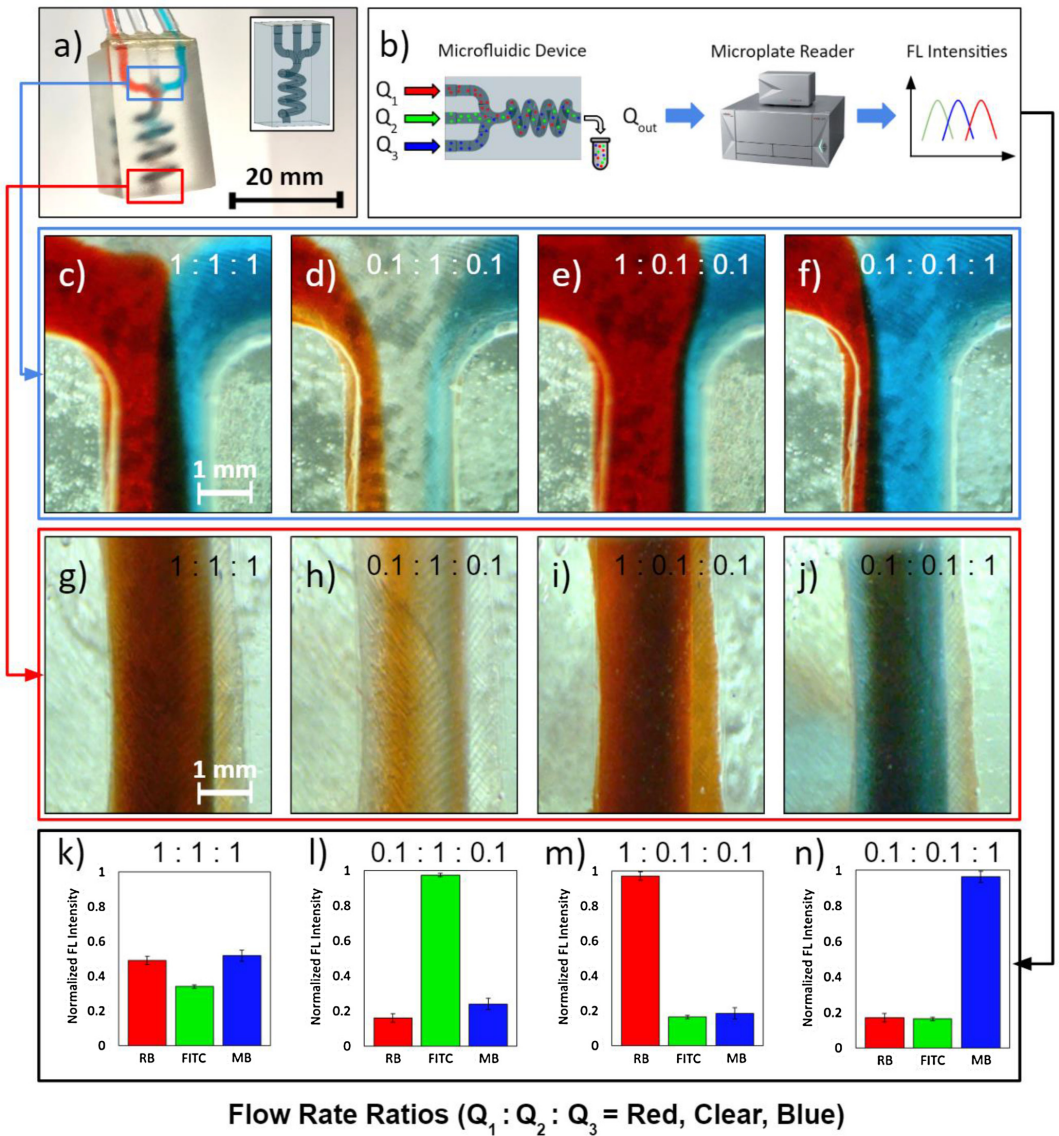
Mixing characterization of the 3D-printed microfluidic architecture. (a) Photograph of the SLA-printed microfluidic mixing architecture with CAD model in the inset. (b) Schematic of the solution concentration quantification method. (c)–(f) Microscopic images of the 3D spiral chamber's inlet junction under various flow rate ratios of red-dyed, clear, and blue-dyed solutions (Q_1_:Q_2_:Q_3_). (g)–(j) Microscopic images of the 3D spiral chamber's outlet under the corresponding flow rate ratios. (k)–(n) Normalized fluorescence (FL) intensities of rhodamine B (RB), fluorescein isothiocyanate (FITC), and methylene blue (MB) present in the solutions obtained from the outlet. Error bars indicate ±standard deviation (*n* = 3).

The printed microneedle arrays can be seamlessly integrated with microfluidic modules by leveraging the one-step fabrication capability of our approach. To demonstrate the versatility of the printing scheme in rendering complex architectures, we present a custom microfluidic module design [shown in Fig. 4(a)], which can be exploited to perform hydrodynamic mixing. This design includes three separate inlet microfluidic channels that converge into a single 3D spiral chamber, where the injected fluids are hydrodynamically mixed with desired ratios (by modulating the injected flow rates) and emerge homogenized at the outlet. The 3D spiral chamber implements a longer overall length than an equivalent 2D rendition (assuming the same device length), which is advantageous in facilitating longer paths for diffusive mixing and promoting a homogenized distribution at the outlet.

We demonstrated multifluid mixing, using the devised 3D spiral chamber, through flow rate modulation tests. Three dye-water solutions were injected through the channels (red-dyed, clear water, and blue-dyed solutions) at different flow rates, and their mixing profiles were analyzed. Here, the flow rate ratios are listed in the following order of solutions: red-dyed, clear water, and blue-dyed (Q_1_:Q_2_:Q_3_). Figure 4(c) shows the inlet junction when the flow rates of each inlet were equal to 100 *μ*l/min (Q_1_:Q_2_:Q_3 _= 1:1:1, serving as a baseline for comparison with the other flow rates). At this flow rate, the onset of mixing and subsequent fully homogenized mixing were observed at the upstream [Fig. 4(c)] and downstream [Fig. 4(h)], respectively. When two of the three inlets were reduced to 10 *μ*l/min, the upstream flow profiles were shifted in favor of the stream with the higher flow rate [Figs. 4(d)–4(f)]. As a result, the fluid with a higher flow rate dominated the downstream solution color [Figs. 4(h)–4(j)].

This effect can be leveraged to tune the outlet solution composition (simply by modulating the injected flow rate at each inlet). To demonstrate this capability, we replaced the original solutions with fluorescent dyes (rhodamine B, fluorescein isothiocyanate, and methylene blue). As illustrated in Fig. 4(b), the fluorescent dye solutions were directly extracted from the device outlet after mixing and analyzed using a microplate reader. Figures 4(k)–4(n) present the fluorescence intensities of each solution (normalized against the maximum intensity of the corresponding dye measured across all the flow rates, for ease of comparison). Microplate readings confirm that the concentrations of the mixtures follow the same trend as that of the input flow rates (i.e., the higher the relative dye solution flow rate, the higher the concentration of the dye at the outlet). Overall, the characterization results demonstrate that by modulating the injected flow rates at the inlet, we can tune the composition of the delivered fluid and achieve homogenized mixing of multiple reagents at the outlet.

### *Ex vivo* transdermal drug delivery

Utilizing the ability of our 3D-printing approach to render microneedle and microfluidic features simultaneously, we 3D-printed the hydrodynamic mixing microfluidic module and the fine-tip syringe-shaped microneedle array as a single device to demonstrate *in-situ* drug modulation/delivery. In order to assess the efficacy of our device, we injected rhodamine B, fluorescein isothiocyanate, and methylene blue through the device and into porcine skin as model-drug molecules. These fluorochromes were selected because of their similar molecular weights, comparable diffusion profiles, and distinct excitation and emission peaks.^43–45^ To quantify drug delivery/modulation, we followed previously reported CLSM and image analysis techniques,^46–48^ and analyzed the penetration depth and relative amounts of the injected fluorescent particles.

**FIG. 5.**
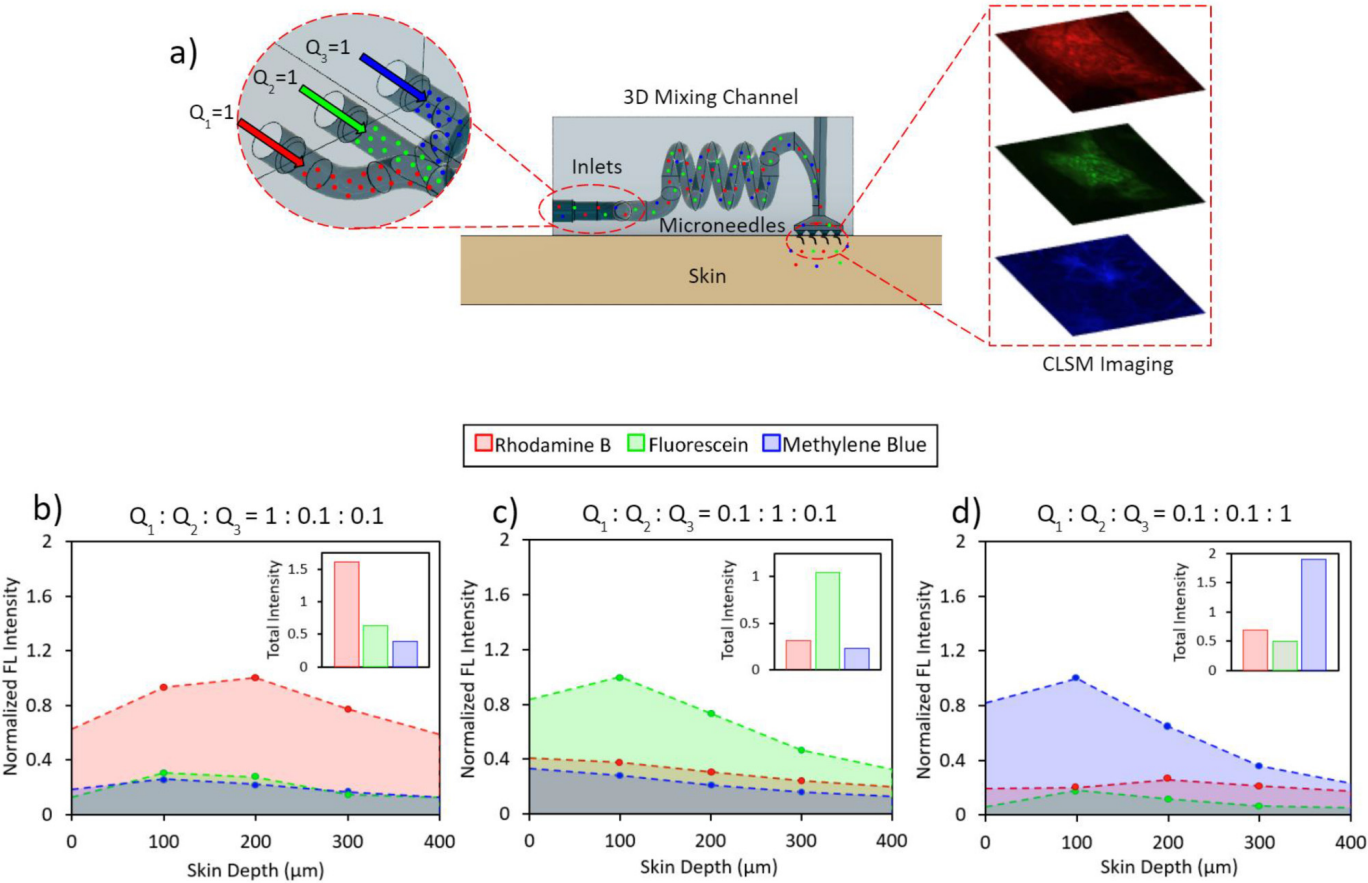
*Ex vivo* transdermal model-drug delivery characterization. (a) The schematic diagram of the experimental setup and the CLSM imaging area. Normalized fluorescence (FL) intensities from CLSM imaging of rhodamine B (RB), fluorescein isothiocyanate (FITC), and methylene blue (MB) on porcine skin at (b) 0.1:0.1:1, (c) 0.1:1:0.1, and (d) 1:0.1:0.1 flow rate ratios across 0–400 *μ*m skin depths (normalized with respect to the 1:1:1 baseline). Insets show the total fluorescence intensity of each dye (the area under the normalized intensity vs the skin depth curve) for the corresponding injection conditions.

Figure 5(a) shows the CLSM experimental setup. The device was placed on a piece of porcine skin (2 × 2 cm) and a 500 g weight atop the device. Then, the same inlet flow rate ratios from the previous mixing experiment were applied, while lowering the actual injected flow rates by a factor of 10 to minimize leakage. The device was then removed from the porcine skin, and CLSM was performed on the microneedle penetration sites. CLSM images were taken at various skin depths, using wavelength-specific excitation lasers and emission filters to examine the fluorescence of each fluorochrome (shown in Figs. S5–S8 in the supplementary material). Figures 5(b)–5(d) illustrate the normalized fluorescence intensities of the CLSM images at each flow rate ratio (see Materials and Methods). The insets of Figs. 5(b)–5(d) present the cumulative fluorescence intensities of the characterized skin depths (0–400 *μ*m), as a quantitative proxy measure of the total amount of each dye transdermally delivered. The raw measured fluorescence intensities of the 1:1:1 baseline flow is shown in Fig. S9 in the supplementary material. Compared to the 1:1:1 baseline, fluorochromes with higher flow rates exhibited a significantly increased fluorescence signal (indicating larger transdermally delivered amounts), which is aligned with the trend observed in the previous microfluidic characterization. Additionally, the imaged fluorescence intensities were highest between 100 and 200 *μ*m, indicating that the fluorochromes were able to reach penetration depths of at least 100 *μ*m, which is aligned with penetration test results from Fig. 3. In summary, the *ex vivo* skin characterization results demonstrate the device's potential for on-demand and programmable drug delivery via modulation of the input fluid solutions' flow rates.

## CONCLUSIONS

Here, we presented an SLA-based 3D-printing scheme to render integrated microfluidic-enabled hollow microneedle devices for transdermal drug delivery. Scanning electron microscopy showed the accuracy, consistency, and repeatability of the 3D-printing scheme across multiple hollow microneedle array designs. Penetration and fracture tests confirmed the microneedles' mechanical robustness for practical application. An example microfluidic-enabled microneedle device was printed with our devised scheme that facilitates homogeneous mixing of multiple fluids under different flow rates, followed by transdermal delivery of the mixed solution. Comparisons of various flow rate ratios with colored dye solutions showed tunable control over the relative concentrations of solutes delivered. *Ex vivo* confocal laser scanning microscopy of three fluorochrome model-drug solutions on porcine skin further validated the platform's ability for transdermal drug modulation and delivery.

This 3D-printed device is particularly applicable to preclinical investigations centered on combinational drug therapy, where the *in-situ* combination of multiple drugs and the tuning of their physicochemical properties lead to more effective outcomes than single or premixed agents alone. For example, the controlled multifluidic synthesis of nanoparticles can tune the release mechanisms of various drugs for wound healing applications.^49^ By leveraging the low-cost and multiscale/high speed printing of SLA, while pushing its resolution limits to render fine hollow microneedle features, the devised fabrication scheme presents new device design possibilities and degrees of freedom for transdermal drug delivery.

## SUPPLEMENTARY MATERIAL

See the supplementary material for details on print orientation characterization (Fig. S1), penetration tests on porcine skin (Fig. S2), microneedle mechanical stability and failure (Figs. S3 and S4), model-drug delivery characterization under various flow rates (Figs. S5–S9), false printing effects (Fig. S10), and the hole formation rate (Fig. S11).
